# Alterations in follicular fluid BMP-15 RNA expression in women undergoing controlled ovarian hyperstimulation

**DOI:** 10.3906/sag-2002-208

**Published:** 2020-08-26

**Authors:** Şükriye Derya DEVECİ

**Affiliations:** 1 Fırat University School of Health Sciences, Elazığ Turkey

**Keywords:** Follicular fluid, BMP-15, RNA expression, assisted reproduction, oocyte

## Abstract

**Background/aim:**

Bone morphogenetic protein-15 (BMP-15) is one of the maturation indicators of the ovarian follicular pool. The aim of this study was to investigate the possible difference of follicular fluid (FF) BMP-15 RNA expression among low, normal, and high responder women attending controlled ovarian hyperstimulation-intracytoplasmic sperm injection (COH-ICSI) cycles.

**Materials and methods:**

This cross-sectional study was conducted with 75 FFs of COH-ICSI cycles performed at the IVF Unit of University Hospital. Twenty FF from low response (group 1), 27 FF from normal response (group 2), and 28 FF from high response (group 3) were recruited for the study between September 2014 and February 2015. Cycle parameters were collected from patient files. FF BMP-15 RNA expression was evaluated with real-time polymerase chain reaction analysis. Statistical analysis was done with SPSS 16.0 version (SPSS Chicago, IL, USA).

**Results:**

The mean age, infertility duration, and body mass index (BMI) of patients were 31.1 ± 4.4 years, 7.4 ± 4.5 years, and 25.6 ± 4.1 kg/m^2^, respectively. There was no significant difference among groups for age, infertility duration, and BMI. There was no significant difference among groups for fertilization rate, implantation rate, pregnancy rate, and live birth rate. Among the 3 groups, FF BMP-15 RNA overexpression and lower expression rates were not significantly different. In all groups, overexpression showed dominance. The pregnancy rate was 45% among women with lower expression and the pregnancy rate among women with overexpression was 26% (P = 0.02). BMP-15 overexpression showed impact on becoming pregnant (OR = 3.7, 95% CI = 1.245–11.299, P = 0.019).

**Conclusion:**

In this study, there was no significant difference in FF BMP-15 RNA expression levels among low, normal, and high responder women. However, overexpression of FF BMP-15 RNA showed a negative impact on pregnancy rates of women who attended COH-ICSI cycles.

## 1. Introduction

Assisted reproductive technologies (ART) aim to improve the fertility capacity of infertile couples. Most of the ART interventions such as controlled ovarian hyperstimulation (COH), oocyte collection (OPU), and embryo transfer (ET) are performed on women. Each of these steps has an impact on cycle outcome. The primary outcome of the COH cycle is the collected mature oocyte number [1]. A mature oocyte is the hallmark of COH and is dependent on the interaction between stimulation protocol and ovarian response. The ovarian response is determined objectively with preovulatory follicular development and blood estradiol levels [1]. Recently, researchers investigated the follicular fluid (FF) cytokine and growth factor levels for identifying the possible factors that have an effect on oocyte maturation [2]. The bone morphogenetic protein-15 (BMP-15) is one of the regulator proteins of ovarian folliculogenesis [3,4]. The BMP-15 protein secreted by the oocytes into the ovarian follicles supports the follicular environment for selection and growth [5]. While some researchers did not observe a difference in the parameters of mature oocyte and antral follicle counts among single nucleotide polymorphism carriers of BMP-15 gene [6], other researchers reported a relationship between ovarian hyperstimulation syndrome and BMP-15 single nucleotide polymorphism [7]. FF BMP-15 levels are another investigation topic among researchers for predicting cycle outcomes [8]. In our study, we intended to investigate the possible difference in FF BMP-15 RNA expression levels among women with low, normal, and high responses undergoing the COH+ET cycle.

## 2. Materials and methods

This cross-sectional study was conducted with 75 infertile couples that attended a COH-intracytoplasmic sperm injection (ICSI)-ET cycle at the In Vitro Fertilization Unit of our University Hospital between June 2014 and February 2015. Twenty women with low response (group 1), 27 women with normal response (group 2), and 28 women with high response (group 3) were recruited for the study. Age, body mass index, and COH protocol were matched among the population. The male factor was not included in this study to prevent bias that can arise from embryonic development. All participants were informed about the study and informed signed consent was collected. Local ethics committee approval was obtained.

### 2.1. COH-ICSI-ET procedure

All patients attended the GnRH antagonist cycle after a basal infertility evaluation. COH was started on day (D) 2–3 of the menstrual cycle according to age, D3 follicle stimulating hormone (FSH) level, and antral follicle count (AFC) with recombinant FSH (Gonal-F®, Merck İlaç Ecza ve Kimya A.Ş., İstanbul, Turkey). When the leading follicle reached 12mm in diameter or blood estradiol level reached 300 pg/mL, 0.25 mg cetrorelix (Cetrotide®, Merck İlaç Ecza AŞ, Türkiye) was started and continued until final maturation. When at least three follicles reached 17mm in diameter, recombinant human chorionic gonadotropin (hCG) (Ovitrelle®, Merck İlaç Ecza ve Kimya A.Ş.) was administered for final maturation. Oocyte pick-up (OPU) was performed at 34–36 h after hCG injection. Following oocyte re­trieval, their cumulus cells were removed by exposure to 20 IU/mL hyaluronidase (ART-4007A, SAGE BioP­harma, Pasadena, CA, USA) in HEPES-based medium for 30 s, followed by mechanical pipetting in HEPES-buffered HTF containing 5 mg/mL human serum albumin (ART-3001, SAGE BioPharma). The collected FF samples were centrifuged for 10 min at 2000 × *g* to remove the blood and the remaining fluid was stored at –80 °C until assay. 

The patients were divided into 3 groups according to the collected mature oocyte numbers as follows: group 1 (n = 20): low response group with mature oocyte number lower than 5; group 2 (n = 27): normal response group with mature oocyte number between 5–10; group 3 (n = 28): high response group with mature oocyte number higher than 10.

In embryology, mature oocytes were inseminated by ICSI after cumulus separation. Fertilization was defined as the observation of 2 pronuclei 24 h after ICSI. ET was performed on day 3 with a single cleavage embryo or on day 5 with a single blastocyst. Luteal support was done with daily application of vaginal 8% progesterone gel and intramuscular 50 mg progesterone. Pregnancy was checked with blood beta hCG test 14 days after ET. Blood beta hCG test was repeated 2 days later for confirming the healthy increment. Transvaginal ultrasonography was performed for visualization of the gestational sac 10 days later. Ongoing pregnancy was defined as the presence of more than 24 weeks of gestation with a live fetus. Implantation rate (ImR) was calculated as the ratio of gestational sac number/transferred embryo number. The fertilization rate (FR) was calculated as the ratio of fertilized oocyte number/mature oocyte number. The pregnancy rate (PR) was calculated as the ratio of beta hCG test positivity/transferred embryo number. The live birth rate was defined as live fetus delivery/transferred embryo number. 

### 2.2. BMP-15 RNA expression analysis

#### 2.2.1. RNA isolation, quantification, and qualification

Total RNA from the FFs of each group was iso­lated using 37 °C Trizol (Reagent Invitrogen, 15596018, California, USA) according to the manufactur­er’s protocol. 

#### 2.2.2. Complementary DNA (cDNA) synthesis 

OneScript® Revers Transcriptase cDNA Synthesis Kit [G234, Applied Biological Materials Inc. (abm), Richmond, BC, Canada] was applied for cDNA synthesis. Reverse transcriptase reactions included RNA specimens with purified total RNA, 1 U/μL MultiScribe reverse transcriptase, 0.25 mM each of dNTPs, 0.25 U/μL RNase inhibitor, and 1× RT buffer. The specimens were placed into a thermal cycler and incubated at 25 °C for 10 mins, at 42 °C for 50 mins, and at 85 °C for 5 min, respectively, and were conserved at 4 °C. Table 1 presents the primers of the study [9].

**Table 1:** Sequence details of polymerase chain reaction (PCR) primers used to analyze mRNA expression in follicular fluids.

Gene	Sequence of primers
BMP15	Forward: CAGTCCTCTATTGCCCTTCT
	Reverse: AATGGTGCGGTTCTCTCTA
β-actin	Forward: GGACTTCGAGCAAGAGATGG
	Reverse: AGCACTGTGTTGGCGTACAG

#### 2.2.3. cDNA amplification with real-time polymerase chain reaction (RT-PCR) 

The cDNA fragments produced by reverse transcription were amplified with RT-PCR by the presence of sequence-specific primers. The RT-PCR was done on repeated cycling of 3 steps. After the preparation of the RT-PCR plate, 2 μL of cDNA samples were placed into the wells. For every sample on ice, 5 μL TaqMan Master Mix, 2.5 μL nuclease-free water, and 0.5 μL primer hybridization probe and component amounts were calculated according to the sample size, placed into Eppendorf tubes, and vortexed. Eight μL of the prepared mixture were added to the cDNA samples on the plate and then optic adhesive tape was used to cover the plate. The samples were centrifuged with miniplate spinner for 1 min in order to decrease formed bubbles and to accomplish precipitation. Real-time PCR was applied by using a standard Master Mix PCR kit [Bright Green 2XqPCR Master Mix, Applied Biological Materials Inc. (abm)] protocol on an Applied Biosystems 7500 Fast Thermal Cycler. The 10 μL PCR contained 1 μLRT product, 1× Taq PCR Master Mix, and 0.5 μM Taq Master gene-specific assay mix [Applied Biological Materials Inc. (abm)]. The RT-PCR reactions were carried out at 95 °C for 10 min, followed by 40 cycles of 95 °C for 15 s, and 60 °C for 60 s. All reactions were repeated 3 times. Gene expression levels were assessed by using the Applied Biosystems 7500 real-time PCR system. β-actin was applied as the control gene.

### 2.2. Statistical analysis

Statistical analyses were performed with SPSS 16.0 version (SPSS Chicago, IL, USA). A comparison of continuous variables between groups was done with Kruskal–Wallis analysis according to distribution normality of data; for binary comparisons, the Mann–Whitney U test was performed. Comparison of categorical variables was done with the chi-square test or Fisher’s exact test, where applicable. For investigation of possible relation and interaction, correlation and regression analyses were performed, respectively. P value below 0.05 was accepted as statistically significant. The gene expression profile was determined by means of nonparametric tests that perform comparisons 2 by 2. Differences in the gene expression profile were determined using nonparametric tests with 2 criteria to define the transcripts that had altered the mRNA abundance of the different sample sets: an absolute fold change (FC) of 2.0 or more and P < 0.05. Positive FC values reflect an overexpression and a negative value denotes underexpression. 

## 3. Results

The mean age, infertility duration, and BMI of 75 patients in the study were 31.1 ± 4.4 years, 7.4 ± 4.5 years, and 25.6 ± 4.1 kg/m2, respectively. There was no significant difference between groups for age, infertility duration, and BMI (Table 2). AFC gradually increased from group 1 to group 3. 

**Table 2:** Demographic characteristics of patients.

	Group 1	Group 2	Group 3	P value
Age (years)	32.7 ± 4.5	30 ± 4.7	30.5 ± 3.6	0.15
Infertility duration (years)	7.9 ± 5.5	6.6 ± 4.1	7.6 ± 3.9	0.63
BMI (kg/m2)	25 ± 3.9	25.9 ± 4.2	25.9 ± 4.3	0.75
TSH (mU/L)	1.7 ± 0.5	1.9 ± 0.7	1.9 ± 0.6	0.43
Prolactin (ng/mL)	12.3 ± 4.5	13.3 ± 5	15.6 ± 6.9	0.22
Antral follicle count	5.8 ± 1.7	11.8 ± 2.3	18 ± 3.8	0.00

Note: Values are presented as mean±SD. BMI: Body mass index; TSH: Thyroid-stimulating hormone.

The COH parameters of the groups are presented in Table 3. The collected mature oocyte count (MOC) and fertilized oocyte count were significantly different between groups (P < 0.05). While estradiol level on hCG day and mature and fertilized oocyte numbers gradually increased from group 1 to group 3, the total gonadotropin dose gradually decreased from group1 to group 3. The endometrial thickness on OPU day, progesterone level on hCG day, and stimulation duration were not significantly different among groups. FR, PR, and ImR were not significantly different between groups, and abortion and live birth rates of groups were not significantly different either (Table 3).

**Table 3:** COH and embryology parameters of patients.

	Group 1	Group 2	Group 3	P value
Total oocyte count	2.7 ± 1.5	8.5 ± 2.4	12.7 ± 3.8	0.007
Mature oocyte count	1.8 ± 0.9	5.5 ± 2.3	10.7 ± 3.4	0.005
Two pronucleus count	1.4 ± 0.6	3.7 ± 1.5	6 ± 2.7	0.04
Transferred embryo number	1 ± 0.5	1 ± 0.6	1.1 ± 0.6	0.55
Endometrial thickness on day of OPU (mm)	10.5 ± 1.9	11.3 ± 1.2	10.4 ± 1.8	0.36
FSH total dose (IU)	3010 ± 1110	2045 ± 510	1380 ± 300	0.002
Stimulation duration (day)	10.4 ± 1.4	9.7 ± 1.2	11 ± 1	0.06
Progesterone level on hCG day (ng/mL)	0.7 ± 0.3	0.5 ± 0.3	0.7 ± 0.2	0.36
Estradiol level on hCG day (pg/mL)	820 ± 200	1550 ± 670	2350 ± 695	0.045
Fertilization rate (%)	67	69	76	0.36
Pregnancy rate (%)	66	50	54	0.49
Implantation rate (%)	38	29	33	0.86
Abortion rate (%)	10	3.6	11.1	0.54
Preterm birth rate (%)	-	7.1	-	0.87
Live birth rate (%)	25	14.3	22.2	0.48

Note:Values are presented as mean±SD and percentage. COH: Controlled ovarian hyperstimulation; FSH: Follicle-stimulating hormone; OPU: Oocyte pick-up; hCG: Human chorionic gonadotrophin.

FF BMP-15 RNA expression rate is presented in Table 4 and on the Figure. 

**Table 4:** BMP-15 RNA expression level among groups.

	Group 1	Group 2	Group 3	P value
RNA underexpression (%)	25	25.9	35.7	0.64
RNA overexpression (%)	75	74.1	64.3	0.72

**Figure:**
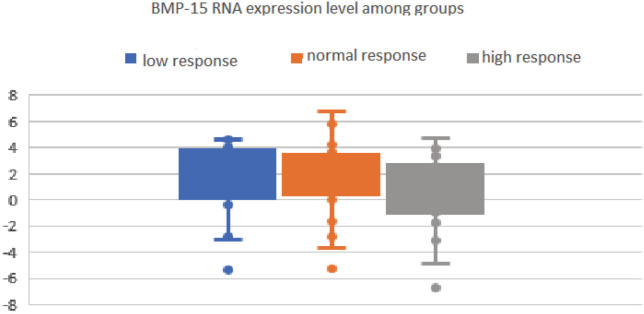
Follicular fluid BMP-15 RNA expression level according to ovarian response to stimulation.

Among the 3 groups, FF BMP-15 RNA overexpression and lower expression rates were not significantly different (Table 4). Overexpression showed dominance in all groups. There was no correlation between FF BMP-15 RNA expression level and the following parameters: age, BMI, estradiol and progesterone levels on hCG day, endometrial thickness on OPU day, FR, PR, and ImR. 

The pregnancy rate of women with either lower or overexpressed BMP-15 RNA is presented in Table 5. The pregnancy rate was 45% among women with lower expression and the pregnancy rate among women with overexpression was 26% (P = 0.02). According to binary logistic regression, BMP-15 overexpression had impact on becoming pregnant (OR = 3.7, 95% CI = 1.245–11.299, P = 0.019). 

**Table 5:** The cross-tabulation between BMP-15 RNA expression and pregnancy rate.

	Pregnancy negative	Pregnancy positive	Total (%)
BMP-15 RNA lower expression (%)	55	45	100
BMP-15 RNA over expression (%)	74	26	100
Total (%)	68	32	100

## 4. Discussion

In this study it was observed that FF BMP-15 RNA expression increased during COH and the expression level did not show a statistically significant difference between low, normal, and high responder women. Low, normal, and high responder women showed similar FR, PR, and ImR. The live birth rate was also similar among the 3 groups. However, women with increased FF BMP-15 RNA expression showed significantly lower pregnancy rates than women with lower FF BMP-15 RNA expression. It is suggested that BMP-15 plays a pivotal role in female fertility [10] and BMP-15 RNA expression is associated with oocyte maturation [11]. Li et al. investigated the cumulus cell BMP-15 mRNA expression level of 196 women suffering from male factor infertility [11]. The stimulation protocol of 196 subjects was a long GnRH agonist protocol. Low responders and high responders were not included in the study. They reported an association between BMP-15 mRNA expression level and age, BMI, and oocyte developmental potential. They observed an association between cumulus cell BMP-15 mRNA expression level and pregnancy outcome. BMP-15 RNA expression level was significantly higher in the group with high-quality embryos than in the group with nonhigh-quality embryos. They observed that pregnant women had higher BMP-15 mRNA levels than women who could not conceive. The results of this study are different from those of Li et al. There was no relation between BMP-15 RNA expression level and age and BMI. And there was a significantly higher level of FF BMP-15 RNA expression in women who could not achieve pregnancy. This difference arises from the study population and design differences. They only investigated normal responder women with male factor infertility, but in this study low, normal, and high responder women without male factor infertility were investigated and pituitary downregulation was done with GnRH antagonist. This is another investigation topic for researchers–the GnRH analog type and its effect on FF BMP-15 RNA expression.

De Resende et al. researched the BMP-15 RNA expression level difference of cumulus cells derived from metaphase II oocytes among 18 women suffering from polycystic ovarian syndrome (PCOS) and 35 controls. Both groups underwent COH with GnRH agonist suppression. They observed significantly higher BMP-15 RNA expression levels in PCOS patients compared to those in controls [12]. In this study, there was no increment on FF BMP-15 RNA expression level of high responder women compared to those in both low and normal responders. This difference may arise from the pituitary downregulation type. De Resende et al. did not observe a correlation between cumulus cell BMP-15 RNA expression level and FF steroid hormone levels [12]. Similarly, in this study, there was no correlation between FF BMP-15 RNA expression level and hCG day serum estradiol and progesterone levels. Liu et al. investigated 5 novel missense mutations of BMP-15 gene in Chinese women suffering from PCOS. They suggested a possible association between BMP-15 gene mutations and PCOS patients [13]. Interestingly, Voorhuis et al. reported a relationship between advanced menopause age and BMP-15 gene single nucleotide polymorphism [14]. Wood et al. observed gene expression differences between high-quality oocytes of normal and polycystic ovaries where morphological discrepancy was not detected [15]. On the other hand, Wei et al. reported decreased BMP-15 expression in PCOS patients that have undergone COH [16]. The contrariety between the study results may arise from differences in PCR (nested vs. real-time) and RNA extraction (Trizol vs. other kits) techniques.

Other researchers experimenting with animals observed increased ovarian BMP-15 expression due to gonadotropin stimulation in rat ovaries [17]. Pennetier et al. researched the duration of bovine oocyte BMP-15 expression from ovulation to the embryonic 8-cell stage; they observed that BMP-15 transcription persisted until embryonic genome activation [18]. Machado et al. investigated the effect of BMP-15 on cattle oocyte during in vitro maturation. They observed an enhancement in oocyte maturation and embryo quality with BMP-15 support [19]. Inagaki and Shimasaki reported an increased ovulation rate with decreased BMP-15 protein production in ewes. Interestingly, they suggested that an increased ovulation rate brings dizygotic twinning, but earlier consumption of ovarian reserve may bring primary ovarian insufficiency [20]. 

Gode et al. reported no relation between BMP-15 expression status and embryo quality on 81 infertile patients undergoing COH+ICSI with GnRH agonist suppression [21]. They included infertile couples suffering from male factor, tubal factor, and unexplained infertility in the study. The results of this study partially confirm the results of Gode’s study. A negative impact of BMP-15 RNA overexpression on the pregnancy rate was observed. The mixed infertility etiology, pituitary downregulation with GnRH agonist, and western blot analysis technique may be the reasons for the differences between our study and Gode’s study.

Researchers reported an increased FF BMP-15 level in poor responder patients compared to normal responder patients [22]. Wu et al. compared FF BMP-15 levels with western blot analysis between 207 poor responder patients and 215 normal responder patients undergoing COH+IVF+ET [8]. Interestingly, they did not report a result for comparison of FF BMP-15 levels between poor responders and normal responders. They divided their poor responder population into two groups according to the mean FF BMP-15 level having either a higher value than the mean value or lower value than the mean value. They had observed increased pregnancy and live birth rates in higher FF BMP-15 level group compared to those in the lower FF BMP-15 group. In a similar manner, in this study, there was a difference in pregnancy rate between FF BMP-15 overexpression and lower expression groups. However, the significantly lower pregnancy rate was observed in the overexpressed FF BMP-15 RNA group. This difference may arise from population and RNA analysis differences between this study and Wu’s study. 

The limitations of this study are as follows: 1) the comparison of FF BMP-15 RNA expression level for embryo quality could not be done; 2) BMP-15 gene mutation analysis could not be performed; 3) the study population could be increased with the opportunity of financial support.

In conclusion, significantly lower pregnancy rates were observed on women with overexpression of FF BMP-15 RNA compared to women with lower expression of FF BMP-15 RNA. However, there was no significant difference for FF BMP-15 RNA expression levels among low, normal, and high responder women.

## Acknowledgment

I thank Dr. Remzi Atılgan, Dr. Şehmus Pala, Dr. Ebru Önalan, Dr. Hüseyin Timurkan, and Mustafa Ekinci for their support during this study.
